# Cooking skills and food insecurity

**DOI:** 10.1371/journal.pone.0326435

**Published:** 2025-06-25

**Authors:** Diego Monteza-Quiroz, Andres Silva, Maria Isabel Sactic

**Affiliations:** Escuela de Nutrición y Dietética, Facultad de Ciencias de la Rehabilitación y Calidad de Vida, Universidad San Sebastián, Santiago, Chile; Lusofona University of Humanities and Technologies: Universidade Lusofona de Humanidades e Tecnologias, PORTUGAL

## Abstract

Cooking skills play a relevant role in food security, which encompasses the availability, accessibility, utilization, and stability of food. While previous discussions have mainly focused on accessibility, particularly economic access through food prices and income, this article explores the dimension of food utilization by analyzing the relation between food insecurity and cooking-related variables. We conducted a survey of 106 low-income households in an urban area of Santiago, Chile. Food insecurity was measured using the Food Insecurity Experience Scale (FIES) developed by the FAO. Using principal component analysis, we constructed two indexes reflecting subjective perceptions of cooking skills. We then applied probit models to examine how both subjective and objective cooking skills variables are associated with the probability of experiencing food insecurity. Results show that individuals who can prepare six to ten egg preparations have an 8.4 percentage point lower prevalence of experiencing food insecurity, while those who can prepare more than ten such preparations show a 30.5 percentage point lower prevalence compared to those who can prepare five or fewer. Moreover, our results found a positive prevalence between negative subjective perceptions and food insecurity of 8.8 percentage point. For the first time, this study jointly examines subjective perceptions and self-reported objective measures of cooking skills in relation to food insecurity. We hope this work contributes to expanding the food insecurity discussion beyond economic access and supports the design of food security policies focused on improving cooking aspects.

## Introduction

Food security, as defined by the Food and Agriculture Organization (FAO), comprises four dimensions: availability, accessibility, stability, and utilization [1]. Although availability and accessibility often dominate discussions, especially on issues such as the cost of healthy diets and the prevalence of food deserts [2,3], utilization and stability are equally important for achieving food security, even though they have received less attention in the literature [4]. Utilization focuses on the nutritional quality of food, which depends on proper food storage and preparation practices. In this context, cooking skills become a key yet often overlooked factor in achieving food security. By enhancing the capacity to prepare healthy meals from available ingredients, cooking skills contribute to food sustainability and support global policy goals such as the Sustainable Development Goal 2.1.

Relying solely on the cost of a diet as a proxy for food insecurity can lead to misleading conclusions. While food may be available and affordable, food insecurity can still arise if it is not used effectively within the household, due to differences in cooking skills, food preparation practices, or other aspects of food utilization. While cost remains a central concern, particularly for low-income households, it does not fully capture the complexity of food security. Healthy diets, especially those rich in fruits and vegetables, are essential because they provide key nutrients [5]. However, healthy diets are often more expensive than calorie-dense, less nutritious alternatives, posing economic challenges [6,7]. Cooking skills can help bridge this gap by enabling households to prepare nutritious meals using ingredients that are often more affordable when purchased in unprocessed forms, thereby reducing reliance on costly pre-prepared foods. Cooking practices have also been positively associated with higher fruit and vegetable consumption [8], highlighting the role of utilization in shaping dietary outcomes [9] Moreover, cooking skills are linked to better diet quality, healthier eating habits, and a lower likelihood of food insecurity [10–12]. In this way, cooking skills improve food utilization and help households overcome some economic and nutritional barriers to achieving food security [13].

Cooking skills are a set of capabilities that encompass mechanical, technical, perceptual, conceptual, organizational and academic aspects, which are not limited to the ability to perform specific tasks, but are also influenced by social, economic and contextual factors such as household size and time availability [14,15]. These skills go beyond performing specific tasks and are shaped by social, economic, and contextual factors, such as household size and time availability [16]. While these dimensions capture some objective aspects of cooking, subjective factors also influence how skills are developed and expressed. These refer to a personal and sentimental assessment about cooking related activities, including personal motivation and enjoyment of cooking [17], emotional relationships with food [18], and confidence in one’s ability to prepare meals [19]. These factors can make the difference between negative feelings, such as seeing cooking as a mandatory or laborious task, and positive feelings, such as seeing cooking as a fun and pleasurable activity, which can influence the nutritional quality of home-cooked meals, potentially leading to improved food safety outcomes.

Sociodemographic factors such as age, gender, and education are associated with the development of cooking skills. Research indicates that being male, young, and having minimal or no educational qualifications are associated with a lower cooking skills level [20,21]. These findings may be partly explained by traditional social norms that have historically associated cooking with women’s roles [22,23]. Among children and adolescents, diet patterns not associated with good health, such as frequent snacking and skipping breakfast, are common [24] and may reflect limited involvement in food preparation, contributing to lower skill development. However, early learning of cooking skills has been shown to improve skill retention, confidence, cooking practices, attitudes towards cooking, and overall diet quality [25]. The role of parents is also important in this process. The presence of parents has been associated with better nutritional outcomes in children [26], and households with children tend to show higher levels of adult cooking skills [17], possibly due to parents’ motivation to provide healthy food for their children [27] and their greater involvement in food-related decisions [26].

Understanding who develops cooking skills also requires clarity on how these skills are measured. Robust measurement tools would help for identifying who possesses these skills and where gaps exist and evaluating their potential association with food insecurity. Some studies use self-reported surveys to assess perceived cooking abilities and daily cooking practices. These include knowledge of techniques such as boiling eggs and vegetables, peeling fruit, and filleting fish [21,25]. Other studies focus on the ability to prepare specific dishes, such as soups or fresh salads [28,29]. Self-reported questionnaires are commonly used, as they offer several advantages, including efficiency in data collection, the ability to address sensitive topics and low cost. However, these methods also present several challenges. For example, there is a lack of consistency in the items and scales used to measure cooking and food skills [30]. Moreover, they often suffer from low response rates, provide only a snapshot of behavior, and are prone to socially desirable responses, which can affect the validity of the results [31]. In addition to these limitations, some questionnaires include subjective measures, such as attitudes toward cooking, but these are rarely disaggregated enough to examine their relationship with specific eating behaviors, such as vegetable consumption [32].

Beyond measurement, interventions have aimed to improve cooking skills and their effects on food-related decision-making, particularly among vulnerable populations such as older adults and low-income individuals [33]. These interventions have reported positive outcomes, including increased fruit and vegetable intake, greater cooking confidence, and improvements in dietary habits [34,35]—factors that could potentially influence food insecurity. However, most evaluations focus on behavioral or nutritional changes without directly assessing their impact on food insecurity. Moreover, the tools used in these interventions are often inconsistent and rarely account for the broader structural and contextual barriers that influence cooking practices and food insecurity. These include low income, lack of access to healthy food, limited time to cook, poor health, lack of cooking facilities, energy poverty, as well as gender and resource access issues globally [36,37]. Perceptual factors, such as lack of enjoyment in cooking, can also play a role. While some educational interventions that integrate nutrition and cooking skills have positively affected food security [38], this area remains understudied and methodologically fragmented.

Addressing this gap in measurement and evaluation is also relevant for policy design, as developing or strengthening cooking skills can be considered a form of human capital investment, potentially offering a more sustainable alternative to subsidies, which often have limited long-term effects [39]. Although the potential role of cooking skills in improving food insecurity has been acknowledged, most research has focused on dietary outcomes rather than explicitly assessing food insecurity. To our knowledge, no previous study has quantitatively examined the relation between objective and subjective dimensions of cooking skills and food insecurity. In addition, although several indicators of cooking skills have been proposed, most are highly context-specific, limiting their comparability between settings. In this context, our study uses a neutral questionnaire and a single dataset to examine the association between subjective perceptions and objective measures of cooking skills based on self-knowledge assessment and to analyze their relation with food insecurity.

## Materials and methods

### Data

The dataset used in this study is derived from the first stage of an experimental study conducted to evaluate residential food waste in low- and middle-income households in the Metropolitan Region of Santiago, Chile. Data collection took place in November 2023, during which comprehensive questionnaires were administered to an individual in each household. This article utilizes a subset of this dataset, focusing on household characterization variables, including detailed information about the household head, household composition, and other determinants frequently cited in the literature on food insecurity. These determinants include cooking skills, and food-related behaviors. The de-identified individual-level dataset is included in the Supporting Information.

In this study, in order to participate, subjects needed to sign an informed consent, to be adults (18 years or older) and responsible for household purchases. Although a target group was not intentionally selected regarding their socioeconomic characteristics, given the geographic location in which the survey was applied, the sample was predominantly composed of low- and middle-income households. The district municipality supported the dissemination of the study through community centers, facilitating the participation of households, which was administered and answered in a virtual format. The complete protocol was approved by the Institutional Review Board from the *Universidad Central de Chile*.

In this study, cooking skills are evaluated using a combination of subjective perceptions and self-reported practical abilities related to food preparation. The cooking skills section of the survey, which constitutes our primary focus, includes nine variables. Seven of these reflect subjective perceptions, measured on a scale from zero to ten, where subjects rated their level of agreement with specific statements. The remaining two variables capture practical aspects: subjects reported the total number of dishes they were able to prepare, as well as the number of preparations they could cook using eggs, used here as a proxy for cooking proficiency. We have asked about egg preparations because subjects need to cook eggs to consume them (they cannot be eaten raw, as fruits). Moreover, eggs are well-known worldwide, therefore, subjects may easily remember some egg preparations. In this sense, we argue that egg-preparation can be thought as proxy of cooking skills.

### Model

We analyzed the relation between cooking skills variables and food insecurity using the survey data, employing the statistical software Stata (version 14.2). Given the diversity of variable types, we implemented three distinct models to compare their effects. All models used a probit regression approach, as the dependent variable was the individual-level probability of experiencing food insecurity, based on the Food Insecurity Experience Scale (FIES) methodology developed by the FAO [40]. This globally standardized methodology generates probabilities of experiencing food insecurity, enabling comparisons across studies and contexts. The independent variables in our models were divided into subjective perceptions of cooking skills, the number of dishes subjects could cook, and the number of egg preparations. Additionally, we included a set of socioeconomic covariates as control variables.

In the first model, we focused on cooking perceptions, utilizing seven perception-based variables. Before fitting the probit model, we conducted a Principal Component Analysis (PCA) to identify the components that captured the most variability across these seven variables. Based on the PCA results, we selected the top components and used them as key independent variables analyze the relation of cooking perceptions and food insecurity. The other two models focused on the quantitative cooking skills variables: the number of dishes subjects could cook and the number of egg preparations as a proxy for advanced cooking skills. These models aimed to assess how different aspects of cooking skills were associated with the prevalence of food insecurity. Finally, we analyzed the marginal effects from all three models to compare and interpret the differences between measures of cooking skills and the likelihood of experiencing food insecurity in low-income households.

## Results

Table 1 shows the descriptive statistics of the sample. Most of the respondents were women, with a partner and low or medium income. Even those subjects classified as mid-income, who make between 500 and 1,000 dollars a month, have a relatively low income. Therefore, four out of five subjects of the sample made less than a thousand dollars a month.

**Table 1:** Descriptive statistics (proportions)

Variable	Mean/Proportion	Std. Dev.
**Sociodemographic variables**		
Gender (0 = men, 1 = female)	0.91	0.29
Age	43.91	123.13
Marital status (0 = without partner, 1 = with partner)	0.74	0.44
**Low income (less than 500,000 CLP)**	0.45	–
**Medium income (500,000–1,000,000 CLP)**	0.38	–
**High income (above 1,000,000 CLP)**	0.16	–
Children, number of	1.51	1.77
Elderly, number of	0.24	0.56
**Subjective cooking perceptions (Average score from 0–10)**		
Enjoy cooking	7.59	2.66
Curious person, likes to try new ingredients	7.68	2.57
Cooking is a way to eat delicious, healthy, and reasonably priced food	8.60	2.07
Cooking is slow, laborious, and boring	3.26	2.93
Cooking takes a lot of time, it is a necessity, and I do only what is strictly necessary	4.84	3.50
Cooking is a pleasure, and I could cook less, but I like it	6.45	3.27
Cooking is a pleasure and a necessity, I have to do it, and I do it with pleasure	8.02	2.57
**Objective cooking skills**		
How many dishes can you prepare?		
1 to 5 dishes	0.15	–
6 to 10 dishes	0.26	–
More than 10 dishes	0.59	–
How many preparations can you make with eggs?		
1 to 5 egg preparations	0.34	–
6 to 10 egg preparations	0.30	–
More than 10 egg preparations	0.35	–
**Food insecurity**		
Moderate or severe probability	0.26	0.37
Severe probability	0.10	0.23
**Observations**	106	

Note: Proportions are values between 0 and 1 (rounded to two decimals). For binary variables such as gender and marital status, values represent the share of female individuals (1) and those with a partner (1), respectively. Income categories expressed in Chilean pesos (CLP). Subjective cooking scores range from 0 to 10.

Considering subjective cooking perceptions, from zero to ten, subjects stated pleasure on cooking with an average score equal to seven or more. Consistently, on average, most subjects disagreed regarding negative feelings about cooking. As presented, 59.4% of the subjects stated to know how to prepare ten or more dishes, while 34.9% of subjects stated to know how to make ten or more egg preparations. It is expected that subjects express to know how to prepare more egg preparations than dishes since some egg preparations cannot be called a dish, such as, scrambled eggs and boiled eggs. It may be the case that subjects do not consider an egg-preparation as a dish.

Results from the PCA indicate that Component 1, PC1, is associated with positive cooking perceptions, such as enjoying cooking and viewing it as both a necessity and a pleasure. In contrast, Component 2, PC2, reflects negative perceptions, including the belief that cooking is boring and time-consuming. The proportion of variance explained by each component and their respective loadings are presented in S2 Table in the Appendix. For comparison, S3 Table displays the marginal effects from a probit model estimated using the, unreduced, original variables. Table 2, using food insecurity as binary dependent variable, present marginal effect using there set of variables. Model 1 includes socioeconomic variables and subjective cooking perceptions, Models 2 and 3 include socioeconomic variables and objective cooking variables.

**Table 2:** Cooking skills and food insecurity.

	Marginal effect, Model 1		Marginal effect, Model 2		Marginal effect, Model 3	
	Coef.	95% CI	Coef.	95% CI	Coef.	95% CI
**Sociodemographic variables**						
Age	–0.007^***^	[–0.010, –0.005]	–0.004^***^	[–0.006, –0.002]	–0.004^***^	[–0.006, –0.002]
Medium income	–0.203^***^	[–0.281, –0.125]	–0.160^***^	[–0.252, –0.069]	–0.135	[–0.272, 0.001]
High income	–0.247^***^	[–0.356, –0.137]	–0.240^***^	[–0.301, –0.178]	–0.254^***^	[–0.318, –0.189]
With partner	0.057	[–0.151, 0.266]	–0.049	[–0.164, 0.263]	0.121	[–0.113, 0.356]
Children, number of	–0.010	[–0.026, 0.005]	–0.012^***^	[–0.018, –0.007]	–0.006	[–0.022, 0.010]
Elderly, number of	–0.102^**^	[–0.157, –0.047]	–0.086	[–0.166, –0.007]	–0.012	[–0.101, 0.076]
**Subjective cooking perceptions**						
PC1	0.038	[–0.013, 0.090]	–	–	–	–
PC2	0.088^***^	[0.051, 0.125]	–	–	–	–
**Objective cooking skills**						
6 to 10 dishes	–	–	–0.219	[–0.536, 0.098]	–	–
More than 10 dishes	–	–	–0.141	[–0.472, 0.189]	–	–
6 to 10 egg preparations	–	–	–	–	–0.084^***^	[–0.143, –0.024]
More than 10 egg preparations	–	–	–	–	–0.305^***^	[–0.349, –0.260]

Note: Coefficients (Coef.) are reported with 95% confidence intervals (CI). ^***^p < 0.01, ^**^p < 0.05, ^*^p < 0.1. The table reports marginal effects on food insecurity (binary outcome) after sequential inclusion of explanatory variable groups.

We also asked about the number of dishes and the number of preparations using eggs that the subject knew how to cook. As presented in Table 2, PC2 variable, in contrast to PC1 variable, is associated to a significant effect on food insecurity. In terms of magnitude, PC2, negative subjective perceptions, is associated with 8.8% effect on food insecurity. The number of dishes did not have a significant association with food insecurity, while the number of egg preparations did have a significant association to food insecurity. A subject who knows how to cook six to ten egg preparations has an 8.4 percentage point lower prevalence of experiencing food insecurity compared to someone who can prepare five or fewer. This prevalence decreases even further—by 30.5 percentage points—for those who know how to prepare more than ten egg preparations. As overall results, knowledge on how to make an egg-preparation is significantly associated with a lower prevalence of experiencing food insecurity. In contrast, general knowledge of food preparations did not show a significant association. Additionally, negative subjective perceptions of cooking were associated with an increased likelihood of food insecurity.

## Discussion

The *Ministerio de Desarrollo Social y Familia*, Ministry of Social Development and Family [41], reports that 15.4% of the population in Chile experiences moderate food insecurity, while 3.5% faced severe food insecurity in 2022. In contrast, our analysis found that 25.7% of subjects in the study experienced moderate food insecurity, and 10.0% faced severe food insecurity, reflecting significantly higher prevalence rates. This difference may be explained by the demographic composition of our sample. In our sample, one out of every four subjects did not have a partner (i.e., they were single, divorced, or widowed), with an average of 1.2 children and 0.4 elderly adults per household. Among households led by individuals without a partner, 96.3% was headed by women with at least one child. Therefore, the higher prevalence observed in our sample could be explained by the larger proportion of households with low- and middle-incomes, as well as the relevant presence of single mother households. Research has indicated that female-headed households, especially those with children, face higher levels of food insecurity [42,43].

Given that most subjects were women and came from low-income households — earning less than a thousand dollars per month, it was expected that they would report higher levels of cooking skills. This expectation was supported by previous research showing that women tend to cook more frequently than men [22,23], and that individuals with lower incomes are generally more likely to cook at home compared to higher-income households [44]. Therefore, the relevance and significance of the relation of egg-preparations and food insecurity, it may be less relevant as income arise. It may be the case that subjects are able to assess in a better way a more specific and concrete question, as egg-preparations, rather than a more general concept as food dishes.

In our study, cooking skills were categorized into two groups: objective variables, which measured the ability to prepare specific dishes (e.g., pasta with sauce, rice with sausage, or lentils) and egg preparations, and subjective variables, which captured positive and negative perceptions of cooking. To assess the objective variables, food groups were not considered in a general way, as the preparation of certain foods or specific dishes can vary significantly depending on the cultural context, which could influence the evaluation of cooking skills [21]. However, developing a cooking skills scale that is culturally independent is a challenging task, as noted in the study by Hartmann *et al*. [17]. The analysis of cooking perceptions revealed that having negative perceptions of cooking such as considering it a laborious task, believing it takes too much time, and viewing it as strictly necessary was associated with an increase of food insecurity. People who perceive cooking negatively may opt for ultra-processed food, either due to a lack of time, work schedules, or convenience [13]. Ultra-processed foods often unhealthy, which may contribute to lower food security [45,46]. However, we cannot assess the actual causal mechanism in place, which remains an area for further research.

These negative perceptions also may be influenced by a lack of confidence in one’s ability to prepare meals [19]. Individuals who are uncertain about their cooking skills are less likely to engage in home cooking, often opting for quicker, less nutritious solutions instead. Additionally, factors such as emotional eating [18] or a lack of personal motivation and love for cooking [19] can further challenge this relation. As a result, addressing these negative perceptions could serve as a key intervention strategy. Studies show that subjects involved in educational programs not only improved their technical cooking skills but also gained confidence in cooking [34], as well as in adopting healthier eating habits [35].

Regarding objective cooking skills, the study found that the ability to prepare between six and ten dishes or more was not associated with a decrease in food insecurity. In line with the reviewed literature, our findings are consistent with some previous studies [28,37,47,48], which found no significant relation between cooking skills and food insecurity, attributing the issue instead to structural factors such as income levels in low-income households. However, regarding the results evidenced on the ability to cook egg preparations, to our knowledge, no previous study has reported comparable effect sizes linking egg preparations with food insecurity outcomes measured through the FIES. However, the literature reports some studies linking egg consumption with improved diet and healthy behaviors [49–51]. This could be explained by the fact that eggs are a nutritious and relatively cost-efficient source of animal protein, which can contribute to improving diet quality and reducing food insecurity, particularly when compared to more expensive protein sources [52]. However, the ability to prepare food dishes alone is not enough and the key could lie both in the technique and in the ingredients used in the kitchen [53,54]. The significant relation found may reflect the importance of incorporating accessible and nutritious ingredients, like eggs into the daily diet. This finding aligns with previous research suggesting that preparing meals at home and increasing self-efficacy in the kitchen are related to healthy eating [55].

## Conclusion

From the four food security dimensions, previous research has disregarded food utilization. In this way, it is implicitly assumed that everyone has the same cooking skills. A skilled cook would be able to prepare a wider variety of fresh foods, while offering a healthy diet to his/her household. In the opposite side, an unskilled cook would have to rely on purchasing prepared food, which tends to be more expensive and less healthy. This study aimed to compare alternative measurements of cooking skills and their relation with food insecurity. Our findings suggest that subjective cooking perceptions and objective cooking skills are related to food insecurity in distinct ways. Negative cooking perceptions, such as viewing cooking as laborious or time-consuming, were associated with a higher prevalence of food insecurity. On the other hand, cooking egg preparations, a practical and accessible skill, was associated with lower prevalence of food insecurity. Interestingly, the number of dishes subjects knew how to prepare, in contrast to egg preparations, did not show a significant relation with food insecurity, suggesting that the type of preparation and its practical application may be more relevant than the number of dishes in general.

This study makes a contribution by addressing food insecurity from the dimension of food utilization, a perspective that remains underexplored. Additionally, it incorporates a standardized measure of food insecurity using the FIES scale developed by FAO, which enhances the comparability of the results with other research. Furthermore, the combination of subjective and objective measures of cooking skills, along with the use of principal component analysis and probit models, provides a more comprehensive methodological approach to examining the relationship between cooking skills and food insecurity. However, we would like to view our findings with some caution. Being a cross-sectional study, it does not allow us to establish a causal relation between cooking skills and food insecurity. Furthermore, cooking skills were self reported, which introduces a risk of bias in subjects perceptions of their own cooking skills. The relatively small sample size and focus on a single low income urban area in Santiago limit the generalizability of our findings. However, while this study focuses on cooking skills, the broader concept of food utilization encompasses other factors not explored here. For example, access to clean water for food preparation, nutrition education, or proper storage techniques are practices that could contribute to nutritional quality.

This study provides preliminary insights that may serve as a foundation for future research aimed at exploring causal relation, while disentangle the mechanism that explains how egg-preparation is related to lower food insecurity prevalence. We also recommend that future studies expand the geographic scope to include rural areas and increase the sample size, as well as dietary preferences and restrictions, and incorporate standardized assessments of cooking skills to strengthen the reliability of the findings. Additionally, we suggest including a broader range of variables related to food utilization, given its role in the conceptualization of food insecurity.

Demographic factors, such as household structure and income levels, provide additional context to the observed prevalence of food insecurity. The overrepresentation of low- and middle-income, and single-mother households in the sample, probably explains the higher prevalence compared to national averages, emphasizing the importance of considering socioeconomic and demographic contexts in cooking skills research, given their possible relation to non-observable characteristics such as cultural norms, time constraints, or access to resources.

Overall, these findings have practical implications worth highlighting. While reducing food insecurity primarily depends on structural factors such as household income, developing cooking skills could have a positive impact on reducing food insecurity. Implementing cooking education programs in schools or community centers, particularly those serving low-income households, could be an effective approach. This could be achieved by using readily available, affordable, and high nutritional value, such as eggs, which in turn discourages the consumption of highly processed foods.

## Supporting information

S1 Table Principal component/correlation (PDF)

S2 Table Component loading (PDF)

S3 Table Cooking skills subjective and food insecurity (PDF)

S4 De-identified individual-level dataset (XLS)
