# Supplementary material for: Cooking skills and food insecurity
Source: PLoS One. 2025 Jun 25;20(6):e0326435. doi: 10.1371/journal.pone.0326435 (PMC12193847; doi:10.1371/journal.pone.0326435)
Supplement: S1 Table — (PDF) [file pone.0326435.s001.pdf]

S1 Table. Principal Component/Correlation

Component	Eigenvalue	Difference	Proportion	Cumulative
1	3.224	1.989	0.460	0.460
2	1.235	0.566	0.176	0.637
3	0.669	0.061	0.095	0.732
4	0.607	0.076	0.086	0.819
5	0.531	0.118	0.075	0.895
6	0.412	0.093	0.059	0.954
7	0.319	-	0.045	1.000
