# Supplementary material for: Cooking skills and food insecurity
Source: PLoS One. 2025 Jun 25;20(6):e0326435. doi: 10.1371/journal.pone.0326435 (PMC12193847; doi:10.1371/journal.pone.0326435)
Supplement: S2 Table — (PDF) [file pone.0326435.s002.pdf]

S2 Table. Component Loading

**Cooking Perception**	**Component 1**	**Component 2**
Enjoy cooking	0.443	-0.169
Curious person and I like to try new ingredients or preparations	0.396	0.219
Cooking is a way to eat delicious, healthy, and reasonably priced food	0.316	0.424
Cooking is slow, laborious, and boring	0.283	0.582
Cooking takes a lot of time, it is a necessity, and I do only what is strictly necessary	0.282	0.631
Cooking is a pleasure, and I could cook less, but I like it	0.418	0.041
Cooking is a pleasure and a necessity, I have to do it, and I do it with pleasure	0.459	0.064
