# Supplementary material for: Cooking skills and food insecurity
Source: PLoS One. 2025 Jun 25;20(6):e0326435. doi: 10.1371/journal.pone.0326435 (PMC12193847; doi:10.1371/journal.pone.0326435)
Supplement: S3 Table — (PDF) [file pone.0326435.s003.pdf]

S3 Table. Effect of Cooking Skills Subjective on Food Insecurity

	Marginal Effect	
**Sociodemographic Variables**	**Coef.**	**95% CI**
Age	-0.008***	[-0.010, -0.006]
Medium income	-0.201***	[-0.241, -0.162]
Medium-high income	-0.227***	[-0.375, -0.079]
With partner	0.023	[-0.171, 0.218]
Children	-0.005	[-0.014, 0.002]
Elderly people at home	-0.114***	[-0.194, -0.033]
**Subjective Cooking Perceptions**		
Enjoy cooking	0.004	[-0.017, 0.025]
Curious person and I like to try new ingredients or preparations	0.043***	[0.018, 0.068]
Cooking is a way to eat delicious, healthy, and reasonably priced food	0.013	[-0.017, 0.044]
Cooking is slow, laborious, and boring	0.000	[-0.001, 0.003]
Cooking takes a lot of time, it is a necessity, and I do only what is strictly necessary	0.009***	[0.003, 0.014]
Cooking is a pleasure, and I could cook less, but I like it	0.037***	[0.022, 0.053]
Cooking is a pleasure and a necessity, I have to do it, and I do it with pleasure	-0.007	[-0.026, 0.012]

Note: Coefficients (Coef.) are reported, and standard errors (Std. Err.) are provided in parentheses.
*** $p<0.01$, ** $p<0.05$, * $p<0.1$
